# Interferon alpha therapy for hepatitis C: treatment completion and response rates among patients with substance use disorders

**DOI:** 10.1186/1747-597X-2-4

**Published:** 2007-01-12

**Authors:** Marilyn S Huckans, Jennifer M Loftis, Aaron D Blackwell, Alex Linke, Peter Hauser

**Affiliations:** 1 Northwest Hepatitis C Resource Center, Portland VA Medical Center, Portland, USA; 2 Behavioral Health & Clinical Neurosciences Division, Portland VA Medical Center, Portland, USA; 3 Department of Psychiatry, Oregon Health & Science University, Portland, USA; 4 Department of Behavioral Neurosciences, Oregon Health & Science University, Portland, USA; 5 Department of Internal Medicine, Oregon Health & Science University, Portland, USA; 6 J.E.N.S. Laboratory, Portland VA Medical Center, Portland, USA; 7 Department of Anthropology, University of Oregon, Eugene, USA

## Abstract

**Background:**

Individuals with substance use disorders (SUDs) are at increased risk for hepatitis C viral infection (HCV), and few studies have explored their treatment responses empirically. The objective of this study was to assess interferon alpha therapy (IFN) completion and response rates among patients with HCV who had a history of comorbid SUDs. More data is needed to inform treatment strategies and guidelines for these patients. Using a medical record database, information was retrospectively collected on 307,437 veterans seen in the Veterans Integrated Service Network 20 (VISN 20) of the Veterans Healthcare Administration (VHA) between 1998 and 2003. For patients treated with any type of IFN (including regular or pegylated IFN) or combination therapy (IFN and ribavirin) who had a known HCV genotype, IFN completion and response rates were compared among patients with a history of SUD (SUD+ Group) and patients without a history of SUD (SUD- Group).

**Results:**

Odds ratio analyses revealed that compared with the SUD- Group, the SUD+ Group was equally likely to complete IFN therapy if they had genotypes 2 and 3 (73.1% vs. 68.0%), and if they had genotypes 1 and 4 (39.5% vs. 39.9%). Within the sample of all patients who began IFN therapy, the SUD- and SUD+ groups were similarly likely to achieve an end of treatment response (genotypes 2 and 3, 52.8% vs. 54.3%; genotypes 1 and 4, 24.5% vs. 24.8%) and a sustained viral response (genotypes 2 and 3, 42.6% vs. 41.1%; genotypes 1 and 4: 16.0% vs. 22.3%).

**Conclusion:**

Individuals with and without a history of SUD responded to antiviral therapy for HCV at similar rates. Collectively, these findings suggest that patients who have co-morbid SUD and HCV diagnoses can successfully complete a course of antiviral therapy.

## Background

Patients with co-morbid substance use disorders (SUDs) and psychiatric disorders are at increased risk for hepatitis C virus (HCV) infection and constitute the vast majority of persons with chronic HCV [1-4]. HCV occurs in up to 90% of injection drug users [5]. Relative to the general population, the incidence of HCV is also high among non-injection drug users. In a sample of over 700 non-injection drug users (heroin, cocaine, or crack) the prevalence of HCV ranged from 5% to 29%, depending on age, gender, study location, and drugs used [6]. To date, few studies have been completed that examine whether ongoing injection or non-injection drug use affects the course of HCV infection.

Currently, standard treatment for HCV is combination therapy with pegylated interferon alpha (IFN) and ribavirin [7,8]. However, in addition to its antiviral effects, IFN-based therapies are also associated with a number of adverse effects, in particular neuropsychiatric side effects [9]. Studies suggest that as many as 75% of patients on IFN report one or more psychiatric side effects including depression, anxiety, insomnia, and impaired concentration [10,11], and approximately 20% – 30% meet criteria for IFN-induced major depressive disorder [12,13]. Healthcare providers have been reluctant to treat adults with co-morbid SUDs because of concerns that these neuropsychiatric side effects may increase risk of relapse [14]. Although there is little empirical data, clinicians may also be concerned that individuals with current or active SUDs are more likely to be non-compliant with treatment, thus jeopardizing efficacy [15]. Most physicians withhold antiviral therapy from HCV-infected alcohol or drug users until patients have maintained abstinence for a period of at least six months [16].

As a result of these attitudes, a disproportionately low number of patients with SUDs have participated in clinical trials or have received antiviral therapy for HCV [17]. In one prospective study of 100 patients who were screened for psychiatric illness, SUDs, or serious medical illness, 68 were found ineligible for IFN therapy due to the presence of at least one of these disorders [18]. Similarly, in another study of 557 patients referred to an HCV clinic, 21% were excluded for psychiatric disorders, 14% for current alcohol abuse, and 3.5% for current injection drug use [19]. One large retrospective study conducted in France found that almost one in six patients with HCV did not receive on-going health care following the diagnosis of chronic HCV [20]. Close to 60% of the patients who did not receive follow-up care belonged to the "high-risk lifestyle group," which included patients with a history of nasal or intravenous drug use and 22.8% of this group were patients with current alcohol abuse (defined as > 50 g/day) [20]. These data suggest that initial barriers to treatment may be related to clinician's negative attitudes about SUDs.

There is a lack of evidence that a history of SUDs preclude treatment tolerance or efficacy. Although sample sizes are small, several recent studies have demonstrated adequate viral response rates in injection drug users still actively using [21-24]. Based on a recent review of 10 clinical trials published between 2001 and 2004 concerning antiviral therapy in substance users, the sustained viral response (SVR) and adherence rates were not different from non-drug users with HCV [25]. Other studies suggest that while heavy drinking (> 70 to 80 g/day) has been associated with poor SVR, individuals with a history of mild to moderate alcohol use who abstain for a period prior to and during IFN therapy have SVR rates that are similar to those found in patients without a history of alcohol abuse [26-30]. Less is known about mild to moderate alcohol use during treatment, and larger sample size studies are still needed to examine treatment compliance and response rates in patients with HCV and co-morbid SUD.

The objective of this study was to assess antiviral therapy completion rates as well as end of treatment response (ETR) and SVR among patients with co-morbid SUDs served by a large VA healthcare network in order to provide empirically based data that may facilitate treatment decisions for this underserved patient population.

## Results

### HCV status, antiviral therapy and demographic information

Within the total sample, 3.7% (11,012/297,712) tested positive for HCV. Of those with HCV, 7.4% (815/11,012) received IFN therapy during the study period (1998–2003). Patients on antiviral therapy were predominantly male (96.0%), middle aged (49.8 +/- 5.7 years), and Caucasian (91.8%).

**Figure 1:**
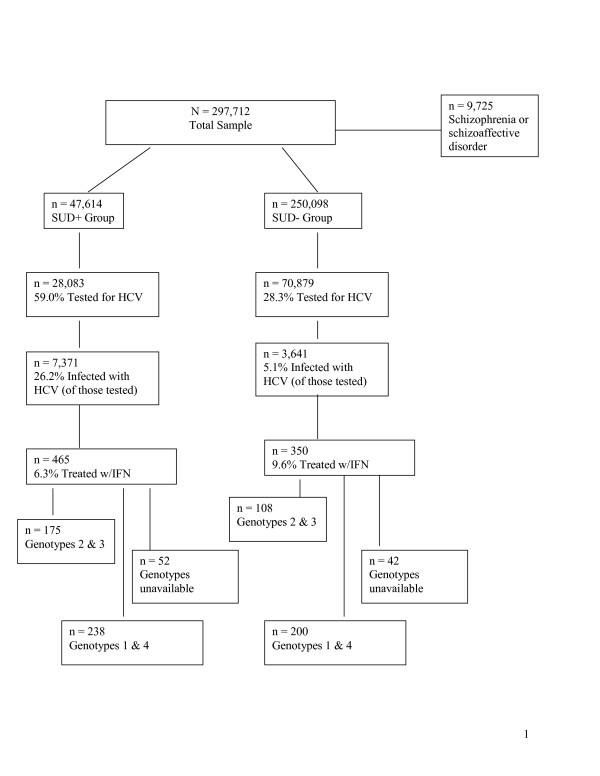
Flow diagram of all study participants. Percentages were calculated based on the number of patients in that group divided by the number of patients in the group one step higher in the flow chart.

Based on SUD diagnoses, patients were categorized into two groups: 1) SUD+ Group: 16.0% (n = 47,614), and 2) SUD- Group: 84.0% (n = 250,098). Figure 1 includes testing, infection, and treatment rates for each group. Table 1 summarizes antiviral therapy completion and response rates for patients who initiated treatment.

**Table 1:** Bivariate relationships between substance use disorders (SUD) and interferon (IFN) completion and response rates among veterans with available hepatitis C (HCV) genotypes

	**SUD- Group**		**SUD+ Group**				
					
	**N**	**%**	**N**	**%**	**OR**	**95% CI**	**p**
**Known vs. Unknown Genotype**							
Not available	42	12.0%	52	11.2%	1.08	0.70–1.67	0.718
Available	308	88.0%	413	88.8%			
							
**Genotypes 2 & 3**							
**Completed ≥ 22 Weeks of IFN**							
No	29	26.9%	56	32.0%	0.78	0.46 – 1.33	0.359
Yes	79	73.1%	119	68.0%			
**End of Treatment Response (ETR)**							
Not available	8	7.4%	9	5.1%	1.38	0.51 – 3.74	0.528
Available	71	65.7%	110	62.9%			
No Response	14	13.0%	15	8.6%	1.56	0.70 – 3.46	0.276
Response	57	52.8%	95	54.3%			
**Sustained Viral Response (SVR)**							
Not available	4	3.7%	8	4.6%	0.79	0.23 – 2.73	0.705
Available	61	56.5%	96	54.9%			
No Response	15	13.9%	24	13.7%	0.98	0.47 – 2.06	0.954
**Response**	**46**	**42.6%**	**72**	**41.1%**			
							
**Genotypes 1 & 4**							
**Completed ≥ 46 Weeks of IFN**							
No	121	60.5%	143	60.1%	1.02	0.69 – 1.49	0.929
Yes	79	39.5%	95	39.9%			
**End of Treatment Response (ETR)**							
Not available	9	8.3%	19	10.9%	0.51	0.22 – 1.21	0.124
Available	70	64.8%	76	43.4%			
No Response	21	19.4%	17	9.7%	1.49	0.71 – 3.13	0.294
Response	49	45.4%	59	33.7%			
**Sustained Viral Response (SVR)**							
Not available	3	2.8%	4	2.3%	1.01	0.22 – 4.69	0.991
Available	55	50.9%	74	42.3%			
No Response	23	21.3%	21	12.0%	1.81	0.87 – 3.79	0.111
**Response**	**32**	**29.6%**	**53**	**30.3%**			

CI = Confidence interval, ETR = End of treatment response, IFN = Interferon, OR = Odds ratio, SUD = Substance use disorder, SVR = Sustained viral response. ORs reflect the SUD+ Group compared with the SUD- Group. The SUD+ Group includes veterans with a history of substance use disorder. The SUD- Group includes veterans with no history of substance use disorder. Completion rates are based on all patients with available genotypes. Response rates are based on all patients with known HCV genotypes who initiated IFN therapy. Patients were considered non-responders if they failed to clear the virus or if they were lost to follow-up (e.g., unavailable ETR or SVR).

HCV genotypes were not available by database or medical record in 11.5% of treated cases, and these cases were not included in subsequent analyses. The SUD+ and SUD- groups did not significantly differ in terms of the percentage of patients with unavailable genotypes.

### Completion and response rate for patients with genotypes 2 and 3

The current standard of care generally recommends for patients with genotypes 2 and 3 to receive 24 weeks of antiviral therapy and for patients with genotypes 1 and 4 to receive 48 weeks of antiviral therapy. Therefore, completion and response rates were analyzed by genotype. For patients with genotypes 2 and 3, antiviral therapy completion rates were not significantly different between the SUD+ and SUD- groups. ETRs and SVRs were unavailable in the electronic medical record database in 6% and 4.2% of cases respectively; the SUD+ and SUD- groups did not differ significantly in the percentage of cases with unavailable ETRs (5.1% vs. 7.4%, respectively) and SVRs (4.6% and 3.7%, respectively). Cases with unavailable ETRs and SVRs were considered lost to follow-up and were considered non-responders in the analysis. Therefore, based on all patients who initiated antiviral therapy (intention to treat analysis), there were no significant differences between the SUD+ and SUD- groups in ETR or SVR (Table 1).

### Completion and response rate for patients with genotypes 1 and 4

For patients with genotypes 1 and 4, antiviral therapy completion rates were not significantly different between the SUD+ and SUD- groups (Table 1). ETRs and SVRs were unavailable in 6.4% and 1.6% of cases respectively; and again groups did not significantly differ in the percentages of unavailable ETRs (10.9% vs. 8.3%, respectively) or SVRs (2.3% vs. 2.8%). Based on intention to treat, there were no significant differences between the SUD+ and SUD- groups in ETR or SVR (Table 1).

## Discussion

Although patients with SUDs have been traditionally excluded from antiviral therapy, our data challenges attitudes that they may not be able to complete or respond to antiviral therapy. There were no significant differences between the SUD + and SUD - groups in completion of 24 weeks of antiviral therapy for genotypes 2 and 3 (73.1%% vs. 68.0%), and in completion of 48 weeks of antiviral therapy for genotypes 1 and 4 (39.5% vs. 39.9%). Additionally, our results indicate that individuals with SUDs have similar ETRs and SVRs as those without SUDs.

To date, our study is the largest sample of HCV patients with co-morbid SUDs (n = 432) that describes completion rates of antiviral therapy. Our results suggest that individuals with a history of co-morbid SUD can successfully complete and respond to antiviral therapy. Furthermore, our results suggest that antiviral therapy is warranted for HCV patients with SUDs.

Historically, patients with HCV and co-morbid SUDs have been excluded from clinical trials. Consequently, IFN therapy completion and response rates for these patient groups are generally unknown. A recent national multi-center study designed to assess the role of alcohol use on HCV treatment outcomes found results similar to those presented in this paper. These investigators found that past alcohol use did not affect the ETR, SVR or discontinuation rates; however, recent alcohol use resulted in higher treatment discontinuation and lower SVR [31]. Although not within the scope of this study, it is possible that patients with co-morbid SUD may need to be more carefully monitored during antiviral therapy in order to avoid adverse effects associated with substance abuse relapse. Future studies that utilize thorough medical record review or prospective design may be able to assess the effect of antiviral therapy on relapse relates as well as what factors may contribute to improved treatment completion and response rates (*e.g.*, co-management by mental health or addiction specialists) among patients with co-morbid SUDs.

Several factors limited the scope and generalizeability of our study. First, results are based on a retrospective review of an electronic medical record database; thus, it is unclear to what extent missing variables, inconsistencies in clinical reporting, or data entry/extraction errors may have affected results. For example, genotypes were unavailable for some patients, and the database lacked accessible ETR and SVR information for many cases. Missing genotype, ETR, and SVR data likely reflect our inability to extract this information from the electronic record when clinicians recorded this information in progress notes rather than in pre-defined database fields. Therefore, a missing ETR or SVR in the laboratory database did not necessarily indicate that a patient was lost to follow-up or that they did not respond. For this reason, response rates were calculated based only on those patients whose genotype, ETR, and SVR were available by database and may be an underestimate of the actual number of patients who achieved ETR or SVR. However, since the methodology and database limitations were equivalent across groups, our conclusion that response rates did not significantly differ between groups, likely remains valid.

Another limitation is the SUD diagnosis. Patients were categorized as having a history of SUD based on inclusion of this diagnosis in their medical records, but the accuracy of these diagnoses could not be confirmed by database. VISN 20 requires and reminds clinicians to complete only a brief substance abuse screening questionnaire with all patients annually, and it is likely that many patients with SUDs remain undiagnosed. It is possible that clinicians varied in their application of diagnostic criteria for SUDs in the electronic medical records. Yet another limitation is the electronic database extraction design, which did not allow us to determine why patients may have discontinued antiviral therapy. It is therefore unclear to what extent important variables (*e.g*., patient noncompliance, side effects or relapse) contributed to discontinuation or non-response. Finally, our veteran sample is primarily Caucasian, middle-aged, and male. Future studies could explore to what extent such demographic variables affect completion and response rates in patients with SUDs.

## Conclusion

Taken together, our results are consistent with emerging research studies and treatment guidelines which suggest that antiviral therapy is feasible in patients with ongoing drug use (other than alcohol), if such patients are likely to comply with treatment and do not have other contraindications [32-34]. This conclusion is consistent with the policy proposed by Edlin et al. which states that; "decisions about the treatment of HCV infection in patients who use illicit drugs be based on individualized risk-benefit assessments, just as they are for other patients. Patient and physician should make decisions about treatment together, after a thorough discussion of the need for adherence to the treatment regimen and the risks of adverse effects and reinfection."[35] In agreement with this policy, the 2002 National Institutes of Health Consensus Statement on the Management of Hepatitis C, the Veterans Health Administration: Treatment Recommendations for Patients with Chronic Hepatitis C, and the 2004 Practice Guidelines for the Management of Hepatitis C recommend that decisions about treatment of HCV in people with psychiatric and SUDs, including injection drug users, be made on a case-by-case basis and advise that drug use itself is not an absolute contraindication to IFN therapy for HCV [36-38].

## Methods

### Sample and data selection

We collected data on 307,437 patients treated between January 1998 and December 2003 at all facilities in the Veterans Integrated Service Network 20 (VISN 20) of the Veterans Healthcare Administration (VHA): 8 medical centers and 17 outpatient clinics in Alaska, Washington, Oregon, and Idaho. Data were extracted from the VISN 20 CHIPS Data Warehouse, a collection of databases from the electronic patient medical records of each facility. We collected data on demographics, psychiatric and substance use diagnoses, HCV laboratory results, and prescriptions. We did not collect data on non-veterans who had records in the system (*e.g.*, employees or family members seen for humanitarian reasons), and so non-veterans were not included in the total sample. The Portland Veterans Affairs Medical Center Institutional Review Board approved data access for this project.

We downloaded data from the VISN 20 data warehouse into a local database using structured query language (SQL) queries, where they were organized and exported to SPSS, version 12.0 for analysis. Patients with a history of schizophrenia or schizoaffective disorder (n = 9,725/307,437; 3.2%), as defined by a Diagnostic and Statistical Manual, Fourth Edition (DSM-IV) code for either disorder in their medical record, were excluded from the total sample and subsequent analyses. Although not well supported by empirical research, it is a common clinical concern that patients with schizophrenia or schizoaffective disorder may have reduced IFN completion and response rates. Since these patients are generally over-represented in SUD populations, we excluded them from further analyses to avoid confounding influences on our results.

### Operational definitions

#### SUD+ group

This group included all patients whose medical record included a history of any SUD as defined by a DSM-IV code for substance abuse or dependence (except nicotine dependence).

#### SUD- group

This group included patients without any documented history of SUD.

#### HCV status

We considered patients to have been tested for HCV if they had at least one HCV lab result in their record between 1994 and 2003 (electronic databases were not reliably extracting data from medical records prior to 1994). HCV positive patients had a positive HCV antibody test, a detectable HCV viral load by polymerase chain reaction (PCR), a positive HCV recombinant immunoblot assay (RIBA), or an identifiable HCV genotype. We classified patients with positive antibody tests but negative RIBA or PCR confirmation as false positives.

#### Antiviral therapy

Antiviral therapy included all types of IFN (*e.g*., IFN-α2a, IFN-α2b, IFN-αn1, IFN-alfacon1, pegylated IFN-α2a, and pegylated IFN-α2b) or combination therapy (IFN and ribavirin) prescribed to patients who were HCV positive. Patients' IFN treatment data were analyzed if records indicated that they had been prescribed at least one prescription for IFN during the study period (1998–2003).

#### HCV genotypes

Genotype lab results were confirmed through electronic medical record review if unavailable in the laboratory database.

#### IFN completion

For patients with genotypes 2 or 3, we considered them to have completed IFN therapy if the period between their first and last IFN prescription release date, plus an additional thirty days (typically the last prescription release is for a one-month period), was greater than or equal to twenty-two weeks. For patients with genotypes 1 and 4, we considered them to have completed IFN therapy if the period between their first and last IFN prescription release date, plus thirty days, was greater than or equal to forty-six weeks.

#### IFN response

We defined an end of treatment response (ETR) as a negative qualitative HCV viral load or a non-detectible quantitative HCV viral load within one month of IFN therapy termination. We defined a sustained viral response (SVR) as a negative qualitative HCV viral load or a non-detectible quantitative HCV viral load at a time point, which was more than six months after IFN therapy termination. Since many patients began therapy before or after the study period (1998–2003), we reviewed lab results from 1994 through June 2006.

### Statistical analyses

Odds ratio analyses were calculated to determine whether patients receiving antiviral therapy (see section 2.2 Operational Definitions) for HCV were less likely to complete or respond to antiviral therapy if they were in the SUD+ Group as compared with the SUD- Group. The database did not allow us to determine whether patients had been recommended six versus twelve months of IFN. However, current standard of care is to prescribe 24 weeks of IFN to patients with genotypes 2 and 3, while individuals with genotypes 1 and 4 are generally prescribed 48 weeks of IFN. Therefore, completion and response rates were analyzed separately for genotypes 2 and 3, and for genotypes 1 and 4. Patients with unknown genotypes were excluded from these analyses. Figure 1 shows how patient groups were selected for analyses.

## Competing interests

The author(s) declare that they have no competing interests.

## Authors' contributions

MSH developed the initial study design, coordinated the statistical analyses, and helped draft the manuscript. JML helped to draft and edit the manuscript and performed some of the statistical analyses. ADB and AL assisted with study design and performed all of the database queries and statistical analyses. PH supervised the study design, provided administrative oversight to this project and edited the final manuscript. All authors read and approved the final manuscript.
